# The Evaluation of Cellular Immunity to Avian Viral Diseases: Methods, Applications, and Challenges

**DOI:** 10.3389/fmicb.2021.794514

**Published:** 2021-12-07

**Authors:** Xiaoli Hao, Fan Zhang, Yi Yang, Shaobin Shang

**Affiliations:** ^1^College of Veterinary Medicine, Yangzhou University, Yangzhou, China; ^2^Institute of Comparative Medicine, Yangzhou University, Yangzhou, China; ^3^Jiangsu Co-innovation Center for Prevention and Control of Important Animal Infectious Diseases and Zoonosis, Yangzhou University, Yangzhou, China; ^4^International Corporation Laboratory of Agriculture and Agricultural Products Safety, Yangzhou University, Yangzhou, China

**Keywords:** cellular immunity, T cells, methods, avian viral diseases, vaccine

## Abstract

Cellular immune responses play critical roles in the control of viral infection. However, the immune protection against avian viral diseases (AVDs), a major challenge to poultry industry, is yet mainly evaluated by measuring humoral immune response though antibody-independent immune protection was increasingly evident in the development of vaccines against some of these diseases. The evaluation of cellular immune response to avian viral infection has long been neglected due to limited reagents and methods. Recently, with the availability of more immunological reagents and validated approaches, the evaluation of cellular immunity has become feasible and necessary for AVD. Herein, we reviewed the methods used for evaluating T cell immunity in chickens following infection or vaccination, which are involved in the definition of different cellular subset, the analysis of T cell activation, proliferation and cytokine secretion, and *in vitro* culture of antigen-presenting cells (APC) and T cells. The pros and cons of each method were discussed, and potential future directions to enhance the studies of avian cellular immunity were suggested. The methodological improvement and standardization in analyzing cellular immune response in birds after viral infection or vaccination would facilitate the dissection of mechanism of immune protection and the development of novel vaccines and therapeutics against AVD.

## Introduction

Immune protection against avian viruses is mainly mediated by adaptive humoral and cellular immunity (Koutsakos et al., 2019b). The level of neutralizing antibody is routinely detected for evaluating immune protection in birds after infection or vaccination. However, antibody-independent immune protection was increasingly evident in the development of vaccines against some of these diseases. For instance, some virus-vectored H7N9 subunit vaccines provided complete protection but did not induce high levels of neutralizing antibodies (Blanchfield et al., 2014; Hu et al., 2017; Stadlbauer et al., 2017; Shi et al., 2019). Clinically, inactivated H9N2 vaccines induced high antibody titers but were unable to completely block the transmission of H9N2 avian influenza virus (AIV) among the immunized flocks (Xu et al., 2018; Bi et al., 2020; Dai et al., 2021). These studies suggest it is necessary to evaluate cellular immune response in order to identify the correlate of immune protection independent of neutralizing antibodies for specific avian viral diseases (AVDs). Indeed, cell-mediated immunity has been shown to play critical roles in the control of avian viral infections. H9N2 AIV-specific CD8 T cells were shown to provide cross-protection against lethal H5N1 infection (Seo and Webster, 2001; Seo et al., 2002). CD3^−^CD8α^+^ NK cells in the lung were shown to be differentially activated by AIV H5N1 and H9N2 infection and correlated inversely with pathogenicity in chicken (Jansen et al., 2013). Depletion of CD8 T cells resulted in increased viral titers of Marek’s disease virus (MDV) in CD4 T cells from CVI988-immunized chickens and more tumor development in monovalent (SB-1 or HVT) and/or bivalent (SB-1 + HVT)-immunized chickens after MDV challenge (Morimura et al., 1998, 1999; Umthong et al., 2020). In addition, cytotoxic CD8 T cells but not CD4 T cells were shown to be responsible for *in vivo* clearance of IBV (Seo and Collisson, 1997; Collisson et al., 2000) and recombinant Newcastle disease virus carrying T cell epitopes from IBV N protein provided significant protection against homologous and heterologous IBV challenge (Qin et al., 2021). Although our understanding of cell-mediated protective immunity to these AVDs was increased, more comprehensive and in-depth analysis of cellular immune responses against avian viral infections is still needed in order to dissect the mechanism of immune protection and develop more effective vaccines.

In general, the investigation of cell-mediated immunity is to address: (1) How each subset of immune cells responds to viral infection or vaccination, including innate and adaptive immune cells such as macrophage, dendritic cells (DCs), NK cell, CD4 and CD8 T cells and so on; (2) How viral antigens are presented by antigen-presenting cells (APC) to activate T cells; and (3) What are the characteristics of the activated T cells and how they mediate immune protection during infection or vaccination (Yang et al., 2020), which are technically involved in the definition of the roles of different immune cell subset, the analysis of T cell functions (activation, proliferation, and cytokine secretion) and *in vitro* culture of APC and T cells for T cell epitope identification. In contrast to our knowledge of cellular immunity against pathogens in human and mouse, we have a very limited understanding of cellular immunity to viral infections in birds. However, with the aid of immunological approaches that are widely applied in the studies of T cell functions and antigen presentation in human and mouse studies (Dai et al., 2019; Yang et al., 2020), the evaluation of cellular immune response in birds after viral infection or vaccination has been realized and increasingly improved through adapted and validated flow cytometry, intracellular cytokine staining (ICS), proliferation assay and enzyme-linked immunospot assay (ELISPOT) that are suitable for birds.

In this review, we summarized the methods that were used for measuring cell-mediated immunity in chickens and their applications in the dissection of cell-mediated immunity against AVD. In addition, the advantages and disadvantages of each method were discussed and potential future directions were suggested to enhance the studies of avian cellular immunity. The methodological improvement and standardization in the analysis of T cell function in chickens would facilitate the dissection of immune protection mechanism and the development of novel vaccines and therapeutics against AVD.

## Flow Cytometric Analysis of Different Cellular Subsets

Flow cytometry is a powerful tool that offers to simultaneously detect the expression of multiple antigens on the surface of or inside a cell and precisely define the phenotypes and functions of distinct immune cell subsets (Soloski and Chrest, 2013). Initially, this technique was used to count absolute numbers of chicken leukocytes in whole blood. As avian erythrocytes and thrombocytes are nucleated and resistant to red blood cell lysis, Burgess and Davison (1999) developed a single-step flow cytometric assay to count absolute numbers of chicken B cells, CD4 and CD8 T cells in whole blood by incorporating fluorescent counting beads, avoiding the separation of leukocytes by density gradient centrifugation and erythrocyte lysis. Using similar strategy, Seliger et al. (2012) established a better flow cytometric assay by combining CD45 and more markers to rapidly enumerate more chicken leukocyte and lymphocyte subsets in whole blood, including thrombocyte, monocyte, T cell, B cell, and heterophilic granulocyte, without prior cell lysis or cell washing steps. Recently, Hofmann and Schmucker (2021) adapted this assay and extended its application for identifying various leukocytes, B cells, and T cell subsets in spleen and cecal tonsils of chickens.

Multi-parameter flow cytometry is particularly useful for acquiring phenotypic and functional data of same sample and widely used in humans and mouse studies (Murdoch et al., 2012; Koutsakos et al., 2019a). However, its application had been hampered in birds due to a paucity of reagents and techniques (Dai et al., 2019; Yang et al., 2020). In recent years, the number of antibodies against chicken cell surface markers has steadily increased, enabling more comprehensive analysis of cellular immune responses following viral infection or vaccination (Table 1). Different avian leukocyte subsets, including monocytes/macrophages, Natural killer (NK) cells, B cells, CD4, CD8, and γδ T cells, have been identified by flow cytometry in the context of infection or immunization (Göbel et al., 2001; Dalgaard et al., 2010; De Boever et al., 2010; Jansen et al., 2013; Taebipour et al., 2017; Larsen et al., 2019). For example, Dalgaard et al. (2010) employed a five-color flow cytometry to assess the frequency of chicken T cell subsets as well as their expression of CD44 and CD45 after Newcastle disease virus (NDV) infection. By a three or four-color flow cytometry, Larsen et al. (2019) examined the dynamic changes of peripheral leukocyte subsets and the expression of cell surface markers in chickens with different MHC-B haplotype after IBV vaccination and found MHC-II expression on monocyte may be a potential correlate of protection. Recently, Dai et al. (2020, 2021) examined host cellular immune response in the peripheral blood of SPF chickens infected with avian leukosis virus (ALV) and H9N2 AIV by polychromatic flow cytometry and found that CD8α^hi^ T cells may participate in ALV clearance (Dai et al., 2020), and CD8 T cell response played important roles in host defense against H9N2 infection (Dai et al., 2021).

**Table 1:** Flow cytometric analysis of leukocyte subsets in chickens after avian virus infection.

Pathogens	Model	Purpose	Major findings	Method	References
NDV	MHC haplotype B21-like, B13 chickens	To assess the frequency of T cell subsets as well as their expression of CD44 and CD45 in peripheral blood from NDV-vaccinated and challenged chickens	Immune chickens from both lines had significant differences in the CD4/CD8 ratio, the frequency of γδ T cells and expression of CD44 and CD45 after vaccination and challenge	Five-color flow cytometry	Dalgaard et al., 2010
IBV	MHC haplotypes B2, B12, B14, B15, B19 and B21 chickens	To examine the dynamic changes of peripheral leukocyte subsets and the expression of cell surface markers in chickens with different MHC-B haplotype after IBV vaccination	MHC-II expression on monocyte might be a potential correlate of IBV vaccine-induced protection	Four-color flow cytometry	Larsen et al., 2019
ALV	SPF chickens	To detect the dynamic changes of T cells in blood and immune organs from chickens infected with ALV	CD8α^hi^ T cells might participate in ALV clearance	Three-color flow cytometry	Dai et al., 2020
AIV	SPF chickens	To detect the dynamic changes of T cells in blood and immune organs from chickens infected with H9N2 AIV	CD8 T cell response played important roles in host defense against H9N2 infection	Three-color flow cytometry	Dai et al., 2021
AIV	SPF chickens	To examine the dynamic changes of myeloid lineage cells, NK cells and T cell subsets in blood and immune organs from chickens infected with H7N9 AIV	H7N9 infection induced distinct local and systemic cellular immune responses in chickens	Six or Seven-color flow cytometry	Hao et al., 2020
MDV	SPF chickens	To detect the dynamic changes of T cell subsets in chickens after immunization with CVI988	CVI988 induced chicken γδ T and CD8α T cells expansion at the early stage after immunization	Six-color flow cytometry	Hao et al., 2021
ILTV	SPF chickens	To evaluate the dynamic changes of CTLs, NK cells and Tregs in the larynx-trachea of pre-immunized with different ILTV vaccines and challenged chickens	Activated CTLs rather than NK cells played a main role in vaccine protection	Six-color flow cytometry	Maekawa et al., 2021

While a basic flow cytometric assay has been used in the abovementioned studies for numerating immune cells, to develop more advanced multi-parameter flow cytometry for analyzing both frequency and function/activation markers of immune cells in chicken is still challenging (Yang et al., 2020). Recently, Hao et al. (2020) integrated most of the antibodies against chicken markers available commercially and developed two panels of 6- and 7-color flow cytometry for comprehensively analyzing chicken myeloid lineage cells, partial NK cells, and T cell subsets as well as their activation. Employing this assay, they found that H7N9 AIV intranasal infection-induced distinct local γδ T and CD8 T cell responses in the lung and systemic NK and monocyte/macrophage responses in chickens (Hao et al., 2020). Using same method, Hao et al. (2021) performed a comprehensive analysis T cell responses to a MD vaccine CVI988/Rispens and revealed the dynamic changes and early activation state of chicken γδ T and CD8 T cells as well as a potential role of γδ T cells in vaccine-induced protection against MD. Recently, Maekawa et al. (2021) also employed a six-color flow cytometric assay to compare the dynamic changes of CD107^+^CTLs, NK cells, and regulatory T cells (Tregs) in the larynx-trachea of chickens immunized with different ILTV vaccines after challenge and found that the cellular immune responses elicited by different ILTV vaccines varied in the upper respiratory tract after challenge, and that activated CTLs rather than NK cells play a main role in vaccine protection. In addition, combining multi-color flow cytometry and gene expression profiling, macrophages and distinct DCs in chickens were characterized and shown to be activated based on upregulated expression of MHC II and costimulatory molecules CD40 and CD80 in the setting of infection (Vu Manh et al., 2014; Meijerink et al., 2021). These studies highlighted the importance to analyze cellular immunity to avian viral infections by flow cytometry. However, the application of flow cytometry in chicken is still limited, compared to its applications in human and rodents. To date, these assays cannot define the phenotype of memory T cells and cytokine production, which are of great importance for identifying correlates of immune protection and the development of effective vaccines in chickens. To develop more advanced multi-color flow cytometry for the evaluation of cellular immunity in chicken may rely on the generation of novel antibodies against chicken markers. The recently-launched Veterinary Immunological Toolbox program^1^ may also help information exchange and sharing of some reagents for this purpose.

## Detection of T Cell Proliferation

The detection of antigen-specific T cell proliferation is one of the hallmarks of lymphocyte activation and differentiation. In poultry, it is essential to assess T cell proliferation for drug screening, vaccine evaluation, and cytotoxicity assessment (Lambrecht et al., 2004; Norup et al., 2011). Classical methods for measuring T cell proliferation include ^3^H-Thymidine (^3^H-TdR) incorporation assays (Maurer, 1981) and MTT assay (Mosmann, 1983) but have disadvantages of generating radioactive waste and lacking of sensitivity, respectively. Therefore, new methods such as CFSE (carboxyfluorescein diacetate succinimidyl ester) and CellTrace Violet (CTV) labelling, BrdU (5'-Bromo-2'-deoxyuridine) and EdU (ethynyl-deoxyuridine) incorporation assays (Motobu et al., 2002; Dalgaard et al., 2010; Alvarez et al., 2020; Meijerink et al., 2021) that are more sensitive and environment-friendly have been introduced for T cell proliferation assay. These methods can be combined with flow cytometry to identify the phenotype of immune cells at the same time and have been validated in chickens (Table 2).

**Table 2:** Flow cytometry-based proliferative assay of T cells in chickens.

Model	Major findings	Method	References
MHC haplotypes B19 and B21 chickens	Live attenuated ND vaccine induced antigen-specific proliferation of CD4, CD8α and γδ T cells upon re-stimulation with inactivated NDV	CFSE assay	Dalgaard et al., 2010
SPF chickens	Identified 4 T-cell epitopes on the S1 protein of IBV that can induce T cells proliferation from pVAX-S1-immunized chickens	CFSE assay	Tan et al., 2016
SPF chickens	Identified 4 T cell epitopes on the N protein of IBV, of which two epitopes stimulated CD8 T cell proliferation and one epitope stimulated both CD4 and CD8 T cell proliferation	CFSE assay	Qin et al., 2021
SPF chickens	BrdU assay can be used to analyze B cells, CD4 and CD8 T cell proliferation after mitogen stimulation and was comparable to ^3^H-TdR incorporation assay in chickens	BrdU assay	Motobu et al., 2002
SPF chickens	EdU assay can reliably distinguish the proliferation of CD4 and CD8 T cells from chicken splenocytes after mitogen stimulation	EdU assay	Alvarez et al., 2020

### CFSE/CTV Labelling Technique

The principle of CFSE labelling is that CFSE, as a cytoplasmic fluorescent dye, reacts with free amine groups of intracellular protein through its succinimidyl ester functional group to form fluorescent protein adducts that can be visualized and quantified by flow cytometry, and thus can be used to track the division and proliferation of the labelled cells (Muul et al., 2011). In human and mouse models, this technique has been widely applied to assess antigen-specific T cell responses (Hoffmeister et al., 2003; Mannering et al., 2005). In addition to its applications in pigs and cattle (Oku et al., 2008; Gerner et al., 2009), this technique has been successfully used in chickens to evaluate B cells (Wattrang, 2009) and T cells proliferation (Dalgaard et al., 2010). Dalgaard et al. (2010) were the first to validate CFSE labelling for the assessment of chicken T cell proliferation. In combination with flow cytometry, they found live attenuated ND vaccine-induced antigen-specific proliferation of CD4, CD8α, and γδ T cells upon re-stimulation with inactivated NDV (Dalgaard et al., 2010). With the aid of this assay, Tan et al. (2016) identified four epitopes on the S1 protein of IBV that can induce T cells proliferation from pVAX-S1-immunized chickens. Moreover, Qin et al. (2021) identified four T cell epitopes on the N protein of IBV, of which three epitopes stimulated CD8 T cell proliferation and one epitope stimulated both CD4 and CD8 T cell proliferation. Although CFSE dye is toxic to cells to some degree and may affect cell viability and modulate the expression of activation markers (Last’ovicka et al., 2009), CFSE labeling is compatible with multi-parameter flow cytometry, facilitating the determination of the phenotypes of the responding cells during proliferation.

Another labelling dye, CellTrace Violet, that is superior to CFSE due to less cytotoxicity and stronger signal has also been employed for T cell proliferation assay in chickens. Using this dye, Lee et al. (2020) found that inactivated H9N2 vaccine adjuvanted with Bacillus subtilis spores induced more proliferative and antigen-specific CD4 and CD8 T cells than did the inactivated H9N2 alone in chickens. This assay has also been used to evaluate the proliferation of CD4 and CD8 T cells following salmonella infection in combination with flow cytometry (Meijerink et al., 2021).

### BrdU/EdU Incorporation Assay

While CFSE and CTV labeling are useful for measuring *in vitro* T cell proliferation, it cannot be used to directly track *in vivo* proliferation of immune cells, especially for those minor subsets that are hard to be cultured *in vitro* (Motobu et al., 2002). BrdU incorporation assay was developed to overcome such a limitation (Reome et al., 2000; Tough et al., 2007), which also avoids the use of radioisotope ^3^H-TdR. BrdU is a thymidine analogue that can incorporate into the synthesized DNA of dividing cells. Through partial denaturation of double-stranded DNA and enzymatic digestion, the incorporated BrdU inside a cell can be exposed and stained by anti-BrdU antibodies to indicate the proliferation of the cells (Bosq and Bourhis, 1997). Lymphocyte proliferation assay *via* BrdU incorporation in combination with multi-color cell surface staining has been widely applied in humans and mice research (Rothaeusler and Baumgarth, 2007). However, this technique has not been applied to assess T cell proliferation in chickens. Although Motobu et al. (2002) developed and validated the use of BrdU incorporation to analyze B cells, CD4 and CD8 T cell proliferation after mitogen stimulation by flow cytometry and showed that this method was comparable to ^3^H-TdR incorporation assay in chickens, this assay has not yet been applied to study chicken lymphocyte proliferation *in vivo* or *in vitro* after infections or vaccination.

As BrdU staining requires denaturation of DNA, digestion with DNase I to expose BrdU and special anti-BrdU antibody, which is time-consuming, an alternative EdU incorporation assay that uses EdU as a thymidine analog was introduced recently (Salic and Mitchison, 2008). This assay is based on a copper-catalyzed reaction that a fluorescent azide is added to an alkynyl group of DNA-incorporated EdU (Salic and Mitchison, 2008) and does not need DNA denaturation and enzymatic digestion, and thus has advantages of faster processing time, extremely bright fluorescence and being compatible with flow cytometry for examining T cell phenotypes and proliferation (Buck et al., 2008; Salic and Mitchison, 2008). This method, as a commercial kit, has been widely applied to human and mouse studies as well as in other mammals but not many in birds (Yu et al., 2009; Sun et al., 2016). Using this method, Warren et al. (2009) examined developmental dynamics of chicken embryo by confocal microscopy. Recently, Alvarez et al. (2020) developed and optimized an EdU assay for analyzing the proliferation of primary chicken splenocytes, which can distinguish the proliferation of CD4 and CD8 T cells by flow cytometry after mitogen stimulation. Although EdU incorporation assay was shown effective for detecting lymphocyte proliferation *in vivo* in mouse (Sun et al., 2016), similar application has not yet been reported in chickens.

## Detection of Cytokine Secretion by T Cells

T cells exert diverse functions in host defense against viral infection. After infection, activated CD8 T cells differentiate into effector T cells, producing cytotoxic granules including granzymes, perforin, and granulysin as well as cytokines such as interferon gamma (IFN-γ) and tumor necrosis factor alpha (TNF-α) to induce programmed death of virus-infected target cells (La Gruta and Turner, 2014). Activated CD4^+^ T cells produce a wide range of cytokines and chemokines (Luckheeram et al., 2012) and differentiate into distinct helper T cell (Th) subsets including Th1, Th2, Th9, Th17, Th22, follicular T helper (Tfh), and regulatory T cells (Treg) based on their expression of cytokines and transcription factors (Beňová et al., 2020). Therefore, it is required to detect the expression of cytokines, transcription factors and degranulation by T cells in order to analyze T cell function and distinguish Th subsets. Besides ELISA, the ICS and ELISPOT assays are the mostly-used approaches to achieve these purposes.

### Intracellular Cytokine Staining

ICS is a peculiarly useful method for detecting the frequency and phenotype of cytokine-producing immune cells. The principle of this method is that cytokines are produced by immune cells upon stimulation with antigens or mitogens and accumulated inside the cells in the presence of an inhibitor, and then the cells are fixed, permeabilized, and intracellularly stained with antibodies against cytokines. Through flow cytometry, the production of cytokines and identities of immune cells are eventually determined (Foster et al., 2007). This assay can be used for simultaneous detection of multiple cytokines and transcription factors expressed by a single cell (Freer and Rindi, 2013), providing functional data about a specific population of immune cells. The ICS assay has been widely used to characterize antigen-specific T cells responses in the context of infection or vaccination in human and mammal studies (Freer and Rindi, 2013; Schmidt and Sester, 2013). However, its application in avian research is still limited. Only a couple of studies employed ICS assay to detect cytokine production by chicken immune cells (De Boever et al., 2010; Huang et al., 2011; Ruiz-Hernandez et al., 2015; Qin et al., 2021). Ariaans et al. (2008) was the first to develop an IFN-γ ICS assay for chicken and used it to detect IFN-γ-producing CD4^+^ and CD8α^+^ T cells after PMA/ionomycin stimulation. De Boever et al. (2010) tried to stain other cytokines including IL-6 and IL-1 β by ICS in chickens but the staining pattern in dot-plots seemed not very convincing. Since then, the IFN-γ ICS assay has been employed to detect antigen-specific IFN-γ-producing T cells in chickens in the trials of attenuated NDV vaccine (Andersen et al., 2017) and IBV epitope-based vaccine (Qin et al., 2021).

Of note, the anti-chicken IFN-γ antibody (clone 5C.123.02) is from a chicken IFN-γ ELISA kit (Invitrogen) and has very weak staining of chicken IFN-γ in the ICS assay (only 2% even upon PMA/Ionomycin stimulation; Ariaans et al., 2008). In our hands, we could not reliably reproduce intracellular staining of IFN-γ following the previous report (Ariaans et al., 2008). Thus, the clone 5C.123.02 may not be an optimal antibody for intracellular staining of chicken IFN-γ. We also tested other 10 clones of anti-chicken IFN-γ antibodies that are commercially available (Table 3), none of them worked for ICS (Hao et al., 2020). A recent study showed that newly-developed six clones of anti-chicken IFN-γ antibodies have strong reactivity with chicken IFN-γ in ICS, which might be a better antibody for intracellular staining of chicken IFN-γ (Lagler et al., 2019). However, in this study, the percentage of IFN-γ was still low (2–3%) and mainly produced by CD4 T cells and barely by CD8 T cells upon mitogen stimulation (Lagler et al., 2019). Thus, it remains debatable whether IFN-γ expression in chickens is just low or the antibodies are not the best. While intracellular staining of IFN-γ and IL-17A have been tested and validated (Walliser and Gobel, 2017, 2018; Lagler et al., 2019), to stain other cytokines intracellularly such as TNF-α and IL-2 has not been reported in chicken. It may be a good option to use quantitative RT-PCR in combination with cell sorting to quantify the expression of IFN-γ and other cytokines by specific subset of immune cells, as shown in a recent study (Hao et al., 2021). Although the Th1-Th2 paradigm in chickens has been described previously (Degen et al., 2005) and chicken αβ and γδ T cells were shown to produce IL-17A (Walliser and Gobel, 2017, 2018), whether other T helper subsets are present in chicken has yet to be discovered. To develop reproducible and reliable ICS for different cytokines may help to identify new Th subsets and characterize T cell functions in chickens after vaccination or infection.

**Table 3:** Chicken IFN-γ antibodies tested in intracellular cytokine staining (ICS) assay.

Antibody ID	Isotype	Conjugate	Source	Catalog number	References
5C.123.08	Mouse, IgG1	Unconjugated	Invitrogen	CAC1233	Lambrecht et al., 2000
5C.123.02	Mouse, IgG1	Biotin	Invitrogen	CAC1233	Lambrecht et al., 2000
MT6C2	Mouse, IgG1	Unconjugated	MabTech	3125-3-250	NA
MT7C10	Mouse, IgG1	Biotin	MabTech	3125-6-250	NA
3E3	Mouse, IgG2a	Unconjugated	In house	NA	Dai et al., 2007
3E5	Mouse, IgG1	Unconjugated	In house	NA	Dai et al., 2007
D8	Mouse, IgG2b	Unconjugated	USCN	MAA049Ga21	NA
Mouse anti chicken interferon gamma	Mouse, IgG	Biotin	USCN	LAA049Ga72	NA
Rabbit anti chicken interferon gamma	Rabbit polyclonal IgG	Unconjugated	Bio-rad	AHP945Z	Weining et al., 1996
Rabbit anti chicken interferon gamma	Rabbit polyclonal IgG	FITC	USCN	LAA049Ga81	NA

FITC, fluorescein isothiocyanate and NA, not applied.

### Enzyme-Linked Immunospot Assay

ELISPOT was originally developed to enumerate antibody-secreting B cells (Czerkinsky et al., 1983). However, it has become a very useful technique to quantify the frequency of cytokine-expressing cells, particularly IFN-γ-secreting T cells in order to evaluate cell-mediated immunity in the setting of infection or vaccination (Sedegah, 2015). This assay displays high sensitivity for detecting cytokine-secreting cells, which can detect as few as one antigen-specific cell per million cells, and is favorable for large-scale screening of antigens or epitopes (Leehan and Koelsch, 2015). From these points, this assay is superior to ICS though it cannot distinguish the phenotype of cytokine-secreting cells unless the effector cells are pre-sorted before plating. IFN-γ ELISPOT assay is one of the most used assays for assessing antigen-specific T cell response in human and mouse studies. This assay has been used in several studies in chickens (Reemers et al., 2012; Tan et al., 2016; Qin et al., 2021). Same to the antibody used for IFN-γ ICS, the anti-chicken IFN-γ antibody pairs (clone 5C.123.08 and 5C.123.02) for ELISPOT are also from the chicken IFN-γ ELISA kit (Invitrogen). Using this pair of antibodies, Ariaans et al. (2009) validated the chicken IFN-γ ELISPOT assay and measured IFN-γ-producing cells in the splenocytes from NDV-vaccinated chickens (Ariaans et al., 2008) and IBV-infected chickens. By this assay, Reemers et al. (2012) performed a large-scale screening of CD8 T cell epitopes on NP and M1 protein of H7N1 AIV that are recognized by different MHC BF molecules (B4, B12, B19) from inbred chicken lines. Recently, IFN-γ ELISPOT assay was employed to identify T epitopes from the overlapping peptides covering IBV S1 protein (Tan et al., 2016) and N protein in chickens (Qin et al., 2021). Through the co-culture of effector cells with infected chicken kidney cell lines, Ruiz-Hernandez et al. (2015) developed a novel co-culture IFN-γ ELISPOT assay which increased the sensitivity, decreased the background of the assay, and enhanced the detection rate of influenza-specific T cell responses in AIV-infected birds. While these IFN-γ ELISPOT assays have contributed to the evaluation of antigen-specific T cell response and epitope mapping in chickens, the weak reactivity of capture antibody (clone 5C.123.08) with chicken IFN-γ may be a concern for the reproducibility of this assay.

## Cytolytic Activity of T-Lymphocytes

Cytotoxic T-lymphocytes (CTLs) play critical roles in the elimination of virus-infected cells and can offer complete protection in instances where antibody immunity is ineffective (Quinones-Parra et al., 2014). CTLs recognize target cells through the interaction of T cell receptor with peptide–MHC complex presented on the surface of the infected cells (La Gruta and Turner, 2014) and induce their programmed cell death *via* directed exocytosis of perforin and granzymes or by the Fas–FasL signaling pathway. The methods for measuring cytotoxicity of CTLs include chromium (^51^Cr) release assay and flow cytometry-based viability assay and CD107a expression. A summary of methods for evaluating cell-mediated cytotoxicity is listed in Table 4.

**Table 4:** Cell-mediated cytotoxicity was measured in avian virus infection.

Virus	Target cell	Effector cell	Major findings	Method	References
MDV	REV-transformed and MDV antigen-transfected cells	Splenocytes of chickens infected with MDV	MHC-restricted, antigen-specific CTL was detected in the spleen of MDV-infected chickens	Cr release assay	Omar and Schat, 1996
MDV	RP9 cells	Splenocytes of chickens infected with MDV	Non-specific cytotoxicity of NK cells was identified in spleen from chickens infected with MDV	Cr release assay	Garcia-Camacho et al., 2003
ALV	The recombinant RP9 cells with MHC class I and ALV antigens	Peripheral lymphocytes from the B21 haplotype chickens inoculated with ALV	MHC-restricted, ALV-specific CTLs were confirmed in the blood of ALV-inoculated chickens	Cr release assay	Thacker et al., 1995
IBV	Chicken kidney cells	Splenocytes from chickens infected with IBV	IBV induces specific CTL activity in chickens	Cr release assay	Seo and Collisson, 1997; Seo et al., 2000
REV	REV transformed B cells	Peripheral lymphocytes from chickens immunized with fixed REV transformed MHC-compatible cells	Specific cytotoxic T cells were detected in the blood of chickens immunized with fixed REV-transformed B cells	Multi-color flow cytometry	Wang et al., 2003
IBV	NT	Lymphocytes from different tissues of IBV-infected chickens	CD107a assay might be a useful tool for measuring cytotoxicity of chicken CTLs	CD107a assay	Wattrang et al., 2015

RP9, LSCC-RP9 (B-cell line); NT, not tested; REV, reticuloendotheliosis virus; MHC, major histocompatibility complex; and CTL, cytotoxic T lymphocytes.

The ^51^Cr release assay has been considered the “gold standard” to measure cell-mediated cytotoxicity. In this assay, the effector cells are generally co-cultured for 4 h at different ratios with target cells that are infected or pre-pulsed with antigen and labelled with ^51^Cr, the release of chromium from the target cells is measured to represent the killing activity of the effector cells. Using this assay, the capability of splenocytes from MD vaccine-immunized chickens to kill reticuloendotheliosis virus (REV)-transformed and MDV antigen-transfected cells and LSCC-RP9 (RP9) cells was measured, which represented MHC-restricted cytotoxicity of the splenocytes and non-specific cytotoxicity of NK cells, respectively (Omar and Schat, 1996; Garcia-Camacho et al., 2003). Similarly, using RP9 cells expressing chicken MHC class I glycoproteins complexed with ALV antigens, Thacker et al. (1995) detected antigen-specific, MHC-restricted CD8^+^CTLs in peripheral blood of ALV-inoculated chickens and found that the difference in cytolytic activity to Rous sarcoma virus (RSV)-induced tumors between B21 and B13 chicken line was associated with their resistance to the tumor development. Using chicken kidney cells as target cells, Seo et al. (2000) found that IBV-specific CTL activity peaked at day 10 after infection and correlated with virus clearance and immune protection (Seo and Collisson, 1997; Seo et al., 2000).

Although the ^51^Cr release assay has extremely high sensitivity, it generates radioactive wastes that are not environmentally friendly, therefore any alternative to this assay is desirable. To this end, Wang et al. (2003) developed a flow cytometry-based assay to measure viral antigen-specific cytolytic activity in chickens, in which the target cells were pre-labelled with a fluorescent dye PKH67 and cell death was detected by propidium iodide staining that labels the DNA of damaged cells. By flow cytometry, they found that specific CTLs were primed after immunization with inactivated, REV-transformed chicken B cells, and the CTL activity can be detected in both CD8^+^ and CD4^+^ T cell subsets (Wang et al., 2003). Using a flow cytometric cytotoxicity assay, Fenzl et al. (2017) found that chicken γδ T cells have spontaneous cytotoxicity to target cells in a non-MHC-restricted manner. As the killing and activation of CTLs *via* perforin-granzyme-mediated pathway is concomitantly associated with transient expression of lysosomal-associated membrane glycoproteins (LAMPs) including CD107a and CD107b on their surface (Betts and Koup, 2004), Wattrang et al. (2015) developed a flow cytometry-based assay to detect the cytotoxicity of chicken CTLs through the assessment of CD107a cell surface mobilization. They found that IBV infection induced the increase of CD107a-positive CTLs in the respiratory tissues of chickens and upon *in vitro* mitogen stimulation, higher proportions of CTLs from IBV-infected chickens showed CD107a mobilization compared to those from naive chickens (Wattrang et al., 2015), suggesting this assay is validated for *in vivo* and *in vitro* detection of chicken CTL cytolytic activity. The availability of these assays may simplify the detection of cytotoxicity and T cell functions to some extent in chickens during avian viral infection and vaccine development.

## *In Vitro* Antigen-Presenting Cell Culture

Dendritic cells bridge innate and adaptive immunity. As professional APCs, DCs play a central role in the initiation of adaptive immune responses, efficiently presenting antigens to T cells. *In vitro* differentiation and culture of DCs is essential for the studies of antigen presentation, DC-T cell interaction, and T cell activation. Chicken DCs were successfully differentiated from bone marrow cells *in vitro* in the presence of recombinant chicken GM-CSF and IL-4 (Wu et al., 2010). These cells displayed high surface expression of MHC class II and CD11c, moderate or low levels of co-stimulatory molecules CD40, CD86, CD83, and DEC205, and can stimulate strong allogeneic mixed lymphocyte responses (MLR; Wu et al., 2010; van den Biggelaar et al., 2020). In chicken, different DC subsets including Langerhans cells (Igyarto et al., 2006), respiratory phagocytes (de Geus et al., 2012) and conventional DCs (cDC; Vu Manh et al., 2014) were also defined by surface markers of putative CD11c (clone 8F2, recently identified as CD11d; Deeg et al., 2020), 74.3, CD83, CD86, MHC-II, KUL01, and DEC205 (Igyarto et al., 2006; Wu et al., 2010; de Geus et al., 2012; Vu Manh et al., 2014; Nagy et al., 2016). However, there is no information on the type and function of DCs in the initiation of adaptive immunity against viral infection in chickens.

Using chicken bone marrow-derived DCs (BMDCs), Liu et al. (2020) examined the effect of H9N2 AIV infection on their transcriptomic profile and found H9N2 virus infection may enhance the signal transduction and innate immune responses but impair their metabolic functions and antigen-presenting responses. Zuo et al. (2021) found that both non-structural protein 7 and 16 of IBV inhibited the maturation and cytokines secretion of BMDCs as well as their abilities to present antigen. Liang et al. (2015) found that inactive IBDV displayed stronger ability than live IBDV to up-regulate the expression of CD40 and CD86 on BMDCs and to stimulate naive T cells proliferation, suggesting live IBDV infection-impaired DC maturation and functions. Vervelde et al. (2013) found that infection of DCs with highly pathogenic (HP) AIV H5N2 and H7N1 but not low pathogenic AIV resulted in increased mRNA expression of IL-8, IFN-α, IFN-γ, TLR1, TLR3 and TLR21, which may be associated with their highly pathogenic nature in chickens. In addition, infection of chicken BMDCs with HPAIV H7N1 also led to increased mRNA expression of DEC205 and macrophage mannose receptor but not DC-SIGN (de Geus et al., 2013). These results suggested that avian viruses with different virulence may modulate the function of chicken DCs. Of note, the yield and phenotype of BMDCs were not well characterized due to the discontinuation of anti-CD11c antibody from Bio-rad in these studies.

In addition to DCs, macrophages and B cells are also capable of presenting antigens in certain circumstance (Wu and Kaiser, 2011). Compared to DCs, macrophages express substantially lower levels of MHC Class I, MHC Class II, and costimulatory molecules even after activation (Trombetta and Mellman, 2005). Chicken macrophages can be prepared from different sources. Early work showed that chicken macrophages can be isolated from peritoneal exudate cells that are induced by injecting Sephadex particles intra-abdominally (Carrel and Ebeling, 1922). In another protocol, chicken monocyte-derived macrophages can be obtained from peripheral blood by adherence to glass or plastic bottom for at least 48 h (Peck et al., 1982). Recently, chicken macrophages were differentiated from bone marrow cells *in vitro* with recombinant chicken CSF1 (M-CSF) or IL-34 (Garceau et al., 2010). Although chicken DCs and macrophages can be cultured *in vitro*, they have seldomly been used as syngeneic APCs to study DC-T cell interaction and T cell activation in chickens after avian viral infection.

## *In Vitro* T Cell Culture

*In vitro* culture of T cells is fundamental for the screening and isolation of antigen-specific CTL clones, functional analysis of T cells, and T cell epitope identification. In human and mouse, antigen-specific T cells can be generally isolated by repeatedly feeding antigen-pulsed APCs and used to identify MHC restriction, CTL activity, and T cell epitopes or antigens, which had contributed to the discovery of novel T cells subsets including peptide-, lipid-, and vitamin B metabolite-specific T cells (Wonderlich et al., 2018). *In vitro* culture of avian T cells has been mainly hindered by the lack of bioactive T cell growth factors, optimal culture conditions, and basic research about the properties of avian T cells. However, a few studies have shown that avian T cells can be cultured *in vitro* (Cihak et al., 1988; Gobel et al., 2003). By cross-linking of Vβ1 T cell receptor (TCR) with the TCR-2 monoclonal antibody, potent proliferation of T cells was induced (Cihak et al., 1988). Chicken IL-2 and IL-18 alone or in combination with TCR activation can also stimulate T cell proliferation, particularly, IL-2 promoted CD8 T cell proliferation whereas IL-18 preferentially induced CD4 growth (Gobel et al., 2003). Using this protocol, chicken CD4 T cells were *in vitro* expanded and used to mimic the transmission of MDV from B cells to CD4 T cells (Schermuly et al., 2015). Although antigen-specific CTLs were detected in many AVD including AIV, IBV, and MDV (Seo and Collisson, 1997; Seo et al., 2002; Dai et al., 2019; Umthong et al., 2020), virus-specific T cell lines or clones have not been isolated and reported in chickens by repeatedly feeding exogenous antigen-pulsed APCs. Recent study showed that *in vitro* culture of avian T cells was feasible in X-VIVO medium in the presence of IL-2 after stimulation with antigen, with low background proliferation, raising a hope for creating CTL clones in chickens (Meijerink et al., 2021). A breakthrough in the isolation of antigen-specific CTL clones may help to address the roles of T cells in anti-viral immunity in chickens.

## Concluding Remarks and Future Perspectives

Cellular immune responses play pivotal roles in the control of viral infection. For AVDs, the evaluation of cellular immune response, especially T cell-mediated immunity has become more and more important as antibody-independent immune protection was constantly observed in the development of vaccines against some of these diseases. With the availability of an array of immunological techniques, which can be used to define different immune cell subsets, examine the activation, cytokine secretion, and proliferation of T cells as well as to culture APCs and T cells *in vitro*, the evaluation of T cell immunity to viral infection in poultry has become feasible and realistic, which would help to dissect the mechanism of cell-mediated immune protection against AVDs and discover novel T cell subsets in birds.

Although the methods for assessing avian cellular immunity, including multi-color flow cytometry, T cell proliferation assay, CTL killing assay, IFN-γ ICS, and ELISPOT assay as well as *in vitro* culture of APCs and T cells, were developed and applied primarily in chickens for the studies of T cell immunity to AVDs, they are suboptimal and not standardized to some extent, compared to similar approaches used in human and mouse. For instance, the number of antibodies against chicken markers is still limited, lacking of antibodies against markers for T cell activation and migration as well as cytokines. The reactivity of anti-chicken IFN-γ antibody currently used for ICS staining is very low as mentioned above. In addition, *in vitro* culture of APCs is not reliably reproduced and the isolation of antigen-specific CTL clones has not been fulfilled in chickens. Therefore, future directions should be to develop immunological tools and optimize those established assays for the evaluation of avian T cell immunity. For instance, developing antibodies against cytokines that are suitable for ICS, expressing recombinant chicken MHC I and II protein to develop tetramer for tracking antigen-specific T cells establishing transfectant expressing single MHC molecules in combination with isolating chicken T cell lines for mimicking *in vitro* antigen presentation. As infectious diseases remain, the major challenge to the poultry industry and antibody-independent immune protection exists, the methodological improvement and standardization in the analysis of T cell function in chickens would help to identify the mechanisms and correlates of immune protection and develop novel T cell-inducing vaccines and therapeutics against AVDs.

## Author Contributions

XH and SS drafted the manuscript. FZ and YY drafted several sections of the manuscript and prepared the table. SS revised the draft. All authors contributed to the article and approved the submitted version.

## Funding

This work was funded by the National Natural Science Foundation of China (32002293), the China Postdoctoral Science Foundation (2018M642345) and a Project Funded by the Priority Academic Program Development of Jiangsu Higher Education Institutions (PAPD).

## Conflict of Interest

The authors declare that the research was conducted in the absence of any commercial or financial relationships that could be construed as a potential conflict of interest.

## Publisher’s Note

All claims expressed in this article are solely those of the authors and do not necessarily represent those of their affiliated organizations, or those of the publisher, the editors and the reviewers. Any product that may be evaluated in this article, or claim that may be made by its manufacturer, is not guaranteed or endorsed by the publisher.

## Footnotes

^1^ https://www.immunologicaltoolbox.co.uk
